# Ethical considerations in digital biomarker adoption

**DOI:** 10.1002/alz70856_097030

**Published:** 2025-12-24

**Authors:** Anna‐Katharine Brem, Emma Poon, Marie‐Christine Fritzsche, Ana Diaz, Dianne Gove, Dag Aarsland, Federica Lucivero, RADAR‐AD Consortium

**Affiliations:** ^1^ King's College London, London, United Kingdom; ^2^ Technical University Munich, Munich, Germany; ^3^ Alzheimer Europe, Senningerberg, Luxembourg, Luxembourg; ^4^ Centre for Age‐Related Medicine, Stavanger University Hospital, Stavanger, Stavanger, Norway; ^5^ Kings College London, London, United Kingdom; ^6^ University of Oxford, Oxford, United Kingdom

## Abstract

**Background:**

Digital biomarkers (DBM) explain and/or predict health outcomes by taking advantage of the advancements in digital technologies and analytics. However, while DBMs offer promising opportunities for research by enabling frequent, remote, real‐time and objective assessments at scale they also raise ethical challenges. Despite growing recognition of these issues, practical guidelines remain scarce. To bridge this gap, we adopt a case study approach by drawing on the ethical challenges encountered in the RADAR‐AD (Remote Assessment of Disease and Relapse – Alzheimer's Disease) project.

**Method:**

RADAR‐AD is a cross‐sectional observational study (*N* = 237) aiming to find and validate remote monitoring technologies to assess cognitive and functional decline in AD. For up to eight weeks, we employed a range of digital technologies, including smartphone apps, wearables and at‐home sensors. This case study addresses two key areas surrounding 1) the ensurance of respect for participants across the research process, and 2) feedback of results and sustainability. By reflecting on the lessons learned in RADAR‐AD, we provide practical recommendations to enhance ethical conduct in DBM research.

**Result:**

The first key area suggests a participatory approach by exploring participants’ needs and concerns in focus groups and involving a patient advisory board (PAB): 1) Informed consent: Explaining the complexities of digital data collection/analysis/storage while engaging large numbers of participants; 2) Burden: Balancing privacy, burden and accessibility during long‐term data collection and offering protocol flexibility; 3) Assistance without being too controlling: Combining phone calls with remote technical checks.

The second key area focuses on 1) Feedback of results: Providing consumer‐grade feedback improves adherence and sense of security and reduces risk of harm; 2) Sustainability: Increasing translation from research to clinical implications by seeking regulatory advice, taking successful results forward to explore in future projects, and sharing data to promote further research. However, contributing tangible results to the healthcare system even after research funding has ended remains a significant challenge.

**Conclusion:**

The RADAR‐AD study provides an exemplary case study highlighting key challenges associated with DBM research and offering valuable insights into ethical considerations that should accompany DBM research from study conception to sustainability efforts.

**Acknowledgment**: The RADAR‐AD project has received funding from the Innovative Medicines Initiative 2 Joint Undertaking under grant agreement No 806999. This Joint Undertaking receives support from the European Union's Horizon 2020 research and innovation programme and EFPIA and Software AG. See www.imi.europa.eu for more details. This communication reflects the views of the RADAR‐AD consortium and neither IMI nor the European Union and EFPIA are liable for any use that may be made of the information contained herein.

